# Exploring Specialist Palliative Care Practitioner Perspectives on the Face Validity of the Attitude to Health Change Scales in Assessing the Impact of Life-limiting Illness on Patients and Carers

**DOI:** 10.1177/08258597211064016

**Published:** 2021-12-13

**Authors:** Linda Machin, Catherine Walshe, Lesley Dunleavy

**Affiliations:** 1 International Observatory on End of Life Care, Lancaster University, Lancaster, UK; 2 School of Primary, Community and Social Care, Faculty of Medicine and Health Sciences, Keele University, Keele, UK

**Keywords:** palliative care, psychosocial support systems, patient outcome assessment, family caregiver, resilience

## Abstract

**Background:** Identifying and assessing vulnerability and resilience through reflexive reactions and conscious coping responses to life-limiting illness is an important, but rarely assessed, component of care. The novel Attitude to Health Change scales can contribute to this, but require fuller development and testing. **Objectives:** Exploring face validity of the Attitude to Health Change Scales (patient and carer versions) from the perspective of specialist palliative care professionals. **Design:** A two-stage study: (i) focus groups to explore experiences of scale use and wording, (ii) online survey to gather preferences on possible scale modifications. Focus group data were analysed using framework analysis. A hermeneutic approach was used to modify the wording of the scales, ensuring adherence to the underpinning concepts used in the design of the scale, congruence with the palliative care context, and simplicity of language. **Setting/Subjects:** Specialist palliative care practitioners in UK hospice settings who had been involved in pilot use of the scales in clinical practice. **Results:** 21 practitioners participated in 3 focus groups across 3 UK hospice sites, 9 of those participants responded to the survey. Four themes are presented: the importance and distinctiveness of the scales; maintaining conceptual integrity; ensuring a palliative care focus; and ensuring linguistic clarity. New iterations of the patient and carer versions of the Attitude to Health Change scales were developed. **Conclusion:** The scales appear to reflect the intended theoretical constructs, and are worded in a way which is congruent with the experience of specialist palliative care practitioners.

## Introduction

Some patients and carers meet the challenges of life-limiting illness with the resilient qualities of courage, perseverance, optimism, and a capacity to make sense of their experience, with adequate support from their network of family and friends.^1^ Others, in the absence of these qualities, are vulnerable to the experience of their unfolding illness.^2^ Differentiating between those patients and carers who are vulnerable will help identify those for whom all aspects of care are likely to be more complex.^3^

Attention to the psychological effect of serious illness is important, but is relatively poorly represented as a core concept in many existing tools.^4^ Some common tools ask questions about care related concerns and symptoms eg IPOS^5^ for patients, or support needs eg CSNAT^6^ for carers. These measures seek to assess the patient's current health circumstances in order to determine an approach to treatment options, care and support. Tools are also available that assess depression, anxiety, distress, and psychological response to cancer.^4,7,8^ Some palliative care practitioners have found that these measures do not go far enough in identifying the underlying factors that determine how well a patient or carer is able to cope with life-limiting illness and its consequences. A distinctive new approach to assessment, not based on pinpointing physical or psychological symptoms, is the Attitude to Health Change self-report scales, one for patients and one for carers. These scales look at the pre-existing, cumulative and invisible factors that shape perspectives on serious illness^9^ such as life experience and personality,^10^ and see these complex interactive personal factors as important for understanding the coping capacity of a patient and their relative degree of resilience and vulnerability. Recognition of the impact of the patient's illness on a family carer is the focus of the carer scale and the implications this has for the complex interaction between patient and carer and the wellbeing of both.^11^ These scales differ from existing palliative care tools in their focus on the underlying individual personal factors which contribute to vulnerability and resilience in both patients and carers. This difference provides insight into the potential capacity to cope effectively, or not, with life-limiitng illness and has implications for providing support where that coping ability is limited or absent. As Grassi et al.^12^ argue,

*‘We do not need further instruments showing reliable properties in comparison with other tested tools, but new instruments that can improve the understanding of psychosocial consequences of medical illness'. ^(p.^ 489^)^*

**Figure 1.**
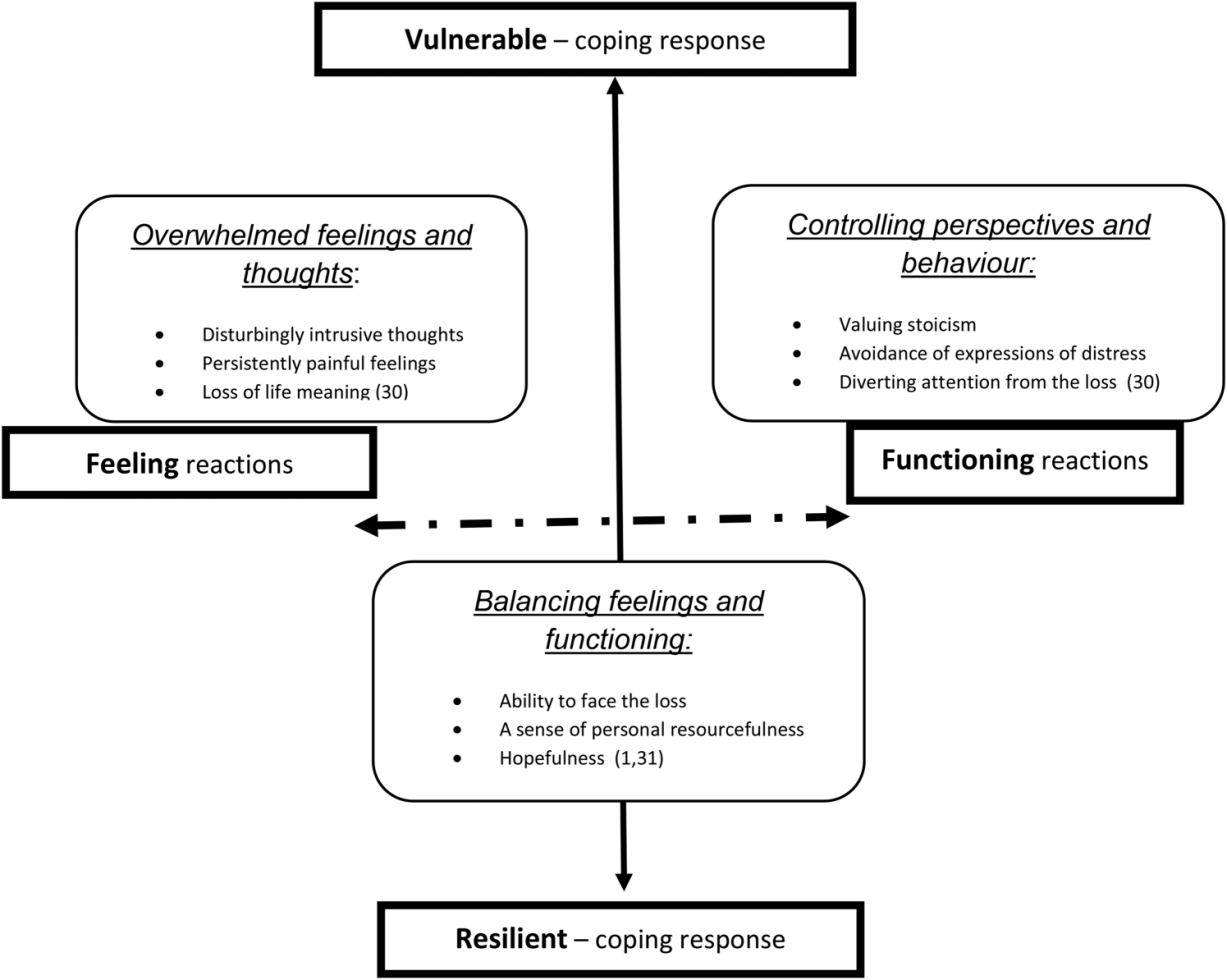
The range of response to loss model showing the intersecting core reactions and coping responses. And the concepts derived from the model represented in the Attitude to Health Change scales.

The concepts that underpin the Attitude to Health Change scales are based on the Range of Response to Loss model,^13^ a new paradigm for conceptualising the nature of loss and its manifestations, crucial for understanding the impact of life-limiting illness for both patients and carers. The model is made up of two interacting dimensions (see table 1 for a description of these dimensions that underpin the tools based on this model, and Figure 1 demonstrating the core reactions and coping responses in the model). The two dimensions interact and “through the scoring system” provide a quantitative measure of vulnerability. The Range of Response to Loss Model also underpins the existing, validated, Adult Attitude to Grief bereavement measure,^14^ which has found traction with practitioners,^15^ and which forms the basis for the development of the new Attitude to Heath Change measures.

**Table 1.** The 2 Interacting Dimensions of the Range of Response to Loss Model.

Firstly, at an instinctive and spontaneous level, **reactions** learned and acquired formally and informally shape the experience and expression of emotion and thoughts. The characteristics of these reactions are described on a spectrum that at one end sees people overwhelmed by their loss and at the other people controlling of their feelings and focused on functioning.
Secondly, at a conscious level people respond to the impact of their loss by a) attempting to balance their feelings and thoughts effectively and b) managing the wider implications of their loss, for example, practical, social, spiritual. These are described as **coping responses** on a spectrum from vulnerable to resilient.

Characteristics of the two dimensions in the Range of Response to Loss model are represented in the 9 items of the Attitude to Health Change scales:

- **Overwhelmed reactions,** are characterised by disturbingly intrusive thoughts, persistently painful emotions and a sense of life losing its meaning^16^ .
- **Controlled reactions** are characterised by a belief in stoicism, avoidance of expression of distress and diverting attention away from what has been or is being lost.^16^
- **Resilient coping responses** are characterised by an ability to face the feelings of loss, a sense of personal resourcefulness to cope with the consequences of loss, and a hopeful and positive sense of being able to accept the loss.^1,17^

The impetus for the development of the Attitude to Health Change scales came from specialist practitioners in palliative care settings, who had successfully used the related Adult Attitude to Grief bereavement measure, and believed that a comparable tool for use with patients and carers would add to the effectiveness of psychosocial assessment and the person-centred support of people facing life limiting illness. An initial ‘Attitude to Health Change’ scale was developed based on the wording of the validated bereavement measure, and used developmentally in practice by a small cohort of specialist palliative care practitioners. They found this intuitively helpful, and identified important factors in its use such as practitioner personal comfort and training; patient and family carer willingness to engage with the scales and having a practitioner “champion” within the organisation.^18^ As part of a planned staged approach to scale development following COSMIN guidelines the aim in this study was to explore the face validity and refine the wording of the Attitude to Health Change scales from the perspective of these specialist palliative care practitioners who have experience of using the emergent scales with patients and family carers

## Materials and Methods

### Purpose and Design

Validity is important in scale development, and face validity, a subset of content validity, is defined as the degree to which items of an instrument reflect the constructs to be measured.^19^ COSMIN guidance recommends that professionals should be asked about the relevance of items, as it is important that the scale has ‘buy-in’ from all stakeholders such that included items are important to clinicians and consistent with the underpinning theory.^20^ The practice context in which a measure is used is an important aspect of developing an understanding of validity21. This perspective was central to the study's purpose and design, drawing from the direct experience of specialist palliative care practitioners use of the developing Attitude to Health Change scales in practice with patients and family carers to explore face validity and suggest refinements to the scales’ wording. Two study methods were chosen: a) qualitative focus groups with hospice practitioners specialising in providing psychosocial support followed by b) an online survey.

### Initial Attitude to Health Change Scales

The items in the Attitude to Health Change scales are theoretically determined, reflecting the two dimensions of the Range of Response to Loss model presented earlier.^13^ The methods used to calculate vulnerability have been validated for use with those who are bereaved in the Adult Attitude to Grief scale which was shown to have construct and discriminative validity.^14^ The proposed 9-item scale covers three categories; controlled functioning, overwhelmed emotion/thinking and resilient coping. Responses to the scales are scored on a five-point Likert scale, from strongly agree to strongly disagree. Vulnerability is calculated quantitatively by combining the overwhelmed and controlled scores with the reversed order of the resilient scores. internal consistency in the three subscales, overwhelmed, controlled and resilient, and that the interconnection between the subscales, support a calculation of vulnerability.

### Population and Setting

Specialist psychosocial palliative care practitioners within UK hospices. Participants were eligible if they were involved in using the Attitude to Health Change scales in practice and/or who worked or volunteered within the hospice in a role which primarily or partly encompassed psychosocial support of patients and their family carers, and where they had experienced others using and discussing the Attitude to Health Change scales.

### Sample

A purposive approach to sampling, focused on known users of the related Adult Attitude to Grief Scale and those using the developmental version of the Attitude to Health Change scale. Hospices known to be using the scales were invited to participate. Following organisational agreement, all those believed to have had experience of using the scales were invited to take part.

### Recruitment

A key contact in each hospice acted as gatekeeper and sent study information to eligible participants. Reply slips were returned to the research team. Written consent was taken from each participant. Those who participated in the focus groups were subsequently invited to participate in the online survey.

### Data Collection

Separate qualitative focus groups were held at each participating hospice.^21^ A topic guide was used to guide but not constrain the discussion, which could iteratively develop. (see Supplemental material one) Participants discussed the underlying theoretical concepts in the Range of Response to Loss model and how far the specific constructs could best be reflected in the Attitude to Health Change scales. Focus groups were audio-recorded and fully transcribed.

Following completion and analysis of focus group(s) data, an online survey was constructed using Qualtrics.^22^ The survey (see Supplemental material two) invited choices based on:

- the scales original wording
- specific suggestions made in the focus groups
- qualitative reflection on retaining theoretically consistency with the Range of Response to Loss model
- simple to understand language
- factors pertinent to life-limiting illness

Participants were asked to state their preferences and give free-text comments on proposed changes.

## Data Analysis

Focus group data were analysed using Framework Analysis, following the process of identifying a framework; indexing; charting; and mapping and interpretation.^23^ The coding framework was iteratively developed through independent coding of transcripts by (L.D.) and (L.M.), with differences resolved through discussion. NVivo was used to develop the framework and manage coding of transcripts. Charts were used to compare and contrast across and between focus groups.

The formulation of survey questions followed the analysis of focus group data and was based on a hermeneutic approach.^24^ Three elements were seen as crucial components in the process of determining wording options presented to respondents and interpreting responses:

1. Ensuring theoretical integrity with the Range of Response to Loss model by consideration of how alignment of the items in the scale to the concepts in the model could be maintained.
2. Identification of wording which was seen as appropriate to variable patient circumstances eg a new diagnosis, changes/deterioration in health, and end of life care.
3. Maximising linguistic clarity by using simple sentences and commonly used words to make the meaning of the items in the scale clear.

Respondents were presented with a number of wording options, generated from the focus group analysis, and with wording considerations based on the principles above, for each of the nine scale items. They were asked to rank their preference for each for both the carer and patient versions of the scale, with an option for free text comment and feedback on wording options presented. Simple numerical preferences were used to determine the preferred options.

## Research Ethics

The study was approved by the Faculty of Health and Medicine Research Ethics Committee at (name removed) (FHMREC18009; 5/10/2018). Each participating organisation gave research governance approval for the study.

## Results

There were 21 participants across three focus groups (see table 2), nine of these participants responded anonymously to the follow up online survey.

**Table 2.** Participant Profiles.

	Site 1	Site 2	Site 3
Total number of participants	10	5	6
Role in hospice	Family support team manager = 1 Social worker/counsellor = 2 Social worker = 1 Counsellor = 2 Spiritual care lead = 1 Physiotherapist = 1 Specialist nurse = 1 Psychologist = 1	Family support team manager = 1 Counsellor = 2 Volunteer = 2	Family services team manager = 1 Counsellor = 3 Volunteer = 2
Practitioner hours of work per week (range)	21 to 37.5 h	3 to 30 h	5 to 32.5 h
Length of experience in hospice work or allied field pertinent to palliative or bereavement care	>5 years = 7 3 to 5 years = 2 <1 year = 1	>5years = 2 3 to 5 years = 1 1 to 2 years = 2	>5 years = 3 3 to 5 years = 2 1 to 2 years = 1
How long individual practitioners have been using the scales (range)	0 to 6 months	2 to 26 months	17 to 48 months
Approximate number of patients and carers individual practitioners have used the Attitude to Health Change scales with	Patients 0 to 10 Carers 0 to 10	Patients 1 to 12 Carers 0 to 10	Patients 1 to 164 Carers 2 to 74

Four themes are presented: the importance and distinctiveness of the scales; maintaining conceptual integrity; ensuring a palliative care focus; and ensuring linguistic clarity. These are followed by more detailed results focused on the precise wording of the scale items.

## Practitioners’ Understanding of the Importance and Distinctiveness of the Attitude to Health Change Scales

Practitioners affirmed the scales relevance to practice and unique nature:

*‘it's not just a detached assessment, it does feel as though there's utility in having a therapeutic conversation with people’. (site 1)*

‘*there is more opportunity to really explore at a deeper level as well which maybe you don't have with other tools’. (site 2)*

Other participants saw the scales providing a framework for holding the diverse agendas of patients and carers:

*‘I think it just asks some very specific questions about all number of things, so that's quite a useful framework to have. It starts a conversation about how they’re managing…whether they’re controlling the situation…how they’re responding to it…gives you a bit more insight into their personal response to illness.’ (site1)*

The effective use of the scales implied positive engagement by patients and carers:

*‘(if) they’re struggling with the language to describe how they’re feeling ……….. (it) gives them a voice in a contained way, ‘ (site 1)*

*‘I handed it to a patient in the first session and we talked about it and she asked if she could take it home, and then she came back to the second session and she really used it amazingly well, she processed a lot of stuff’. (site 1)*

*‘With patients it's (use of the AHC) a really good way of sorting out the nub of what's going on’. (site 3)*

There was clear affirmation that the scales fulfil an important function in quantitatively assessing patients and carers attitudes towards the patient's illness and qualitatively in prompting conversation about those perspectives.

## Maintaining Conceptual Integrity of the Scales

It is sometimes necessary for practitioners to look for words or ideas to which the patient or carer might more readily relate, while retaining the underlying theoretical concepts in the scales. The following gives an example of how a practitioner was able to make an intended meaning clearer to the patient.

Item 3. *‘When thinking about patients being unclear about “inner strength’’ I used an example of when he had to go back to the oncologist to hear more bad news, did he feel he had the strength in himself to go and do that or did he feel the bad news had dented that?* (site1)

This provided the patient with a way of understanding the concept of ‘inner strength’, from his own experience.

The cultural acquisition of notions of ‘being brave’ (item 4) was picked up as important ie how emotional reactions and the meanings attached to them are derived from social learning.

Item 4: *‘This is quite a therapeutic question because often it's where people's belief system is clashing with what they’re able to do, and so actually drawing out some of the discord in themselves is because they’re not able to live by their beliefs anymore or you know they can't function, is often it's quite a good clinical question.’* (site3)

Focus group discussion identified particularly the concepts of ‘inner strength’, ‘being brave’ and ‘making sense of life’ in items 3, 4, and 7 as most likely to need clarification.

## Ensuring a Palliative Care Focus

Practitioners were able to combine their working knowledge of the Adult Attitude to Grief scale and palliative care expertise to offer views about ways to improve the face validity of the Attitude to Health Change scales.

‘*The challenge is also to make the wording here fit the health change context rather than just transpose from the bereavement ones'. (site2)*

An important revision was to replace the repeated use of ‘changes/deterioration in health’ in each item, which participants found ‘*a bit convoluted’* (site 3) or ‘*very cumbersome’* (site 2), with the more generic use of illness/health. This is more simply applicable at any phase of a life-limiting illness.

Two specific items were challenging in the palliative care context. Two focus groups noted that in Item 3 the use of the term “inner strength” could be misunderstood and confused with a physical state rather than a psychological one.

*‘when you have a patient who is quite poorly through treatment and other things, the word ‘strength’ for them is just not, they can't comprehend it.’ (site2)*

Item 8 and the concept of “getting on with life” was seen as problematic in two of the study sites:

*‘if you’re ill you may not have the physical capacity to get on with life‘. (site2)*

While for a carer there may be no choice but to ‘get on with life’.

## Ensuring Linguistic Clarity

The original scales were seen to contain a number of items where clarification would add to their practice usefulness. In Item 1, there was some debate about the word “face” in relation to ‘facing feelings’. There was a suggestion that “cope” might be a better word but the counter to this was,

*‘You could cope with something but not necessarily confront and face it I suppose’. (site2*)

The use of the word “constant” relating to sadness, in item 5 was not liked by several participants. Items 5 and 9 were identified as being too long and introducing several issues that could be confusing. The ideas discussed in the focus groups shaped the options provided in the online survey.

## Survey Results

The wording options derived from the focus group analysis and preference scoring for each of the nine scale items are presented in table 3.

**Table 3.** Attitude to Health Change Scale Wording Options and Responses.

Question	Patient scale options, where option 1 is the original question option.	Number voting for a preference for this version in patient scale	Number voting for a preference for this version in carer scale
1	1. I am able to face the feelings which arise as my health changes/deteriorates.	0	1
	2. I am able to face up to the feelings I have about my illness	5	6
	3. I am able to face up to the feelings I have about my changing health	4	0
	4. No preference.	0	2
2	1. For me, it's difficult to switch off thoughts about the changes/deterioration in my health	1	1
	2. I find it difficult to switch off thoughts about the changes in my health	6	2
	3. I am finding it difficult to switch off thoughts about my illness	2	5
	4. No preference	0	1
3	1. I feel very aware of my inner strength when facing the consequences of the changes/deterioration in my health	1	2
	2. Inside, I have a sense of my ability to cope with my illness and its consequences	3	4
	3. I know I have the inner courage to face the changes in my health	5	2
	4. No preference	0	1
4	1. I believe that I must be brave in the face of my changing/deteriorating health	2	2
	2. I believe I must be brave in facing the changes in my health	4	4
	3. It is my belief that I should be brave in facing my illness	4	2
	4. No preference	0	1
5	1. I feel a constant sense of sadness about the losses caused by the changes/deterioration in my health	3	1
	2. I feel persistently troubled (eg sad, anxious) about my health	3	5
	3. I am persistently overwhelmed by feelings about my illness	0	1
	4. I am emotionally overwhelmed by the changes my illness is bringing	2	1
	5. No preference	0	1
6	1. For me, it is important to keep feelings about my changing /deteriorating health under control	5	4
	2. It is important for me not to react emotionally to the changes in my illness	3	2
	3. I react to my illness by shutting off my feelings	1	2
	4. No preference	0	1
7	1. My changing/deteriorating health makes it harder for me to make sense of life	1	2
	2. My illness and the changes I am facing make it harder for me to make sense of life	2	1
	3. This illness makes me wonder what life is all about	2	3
	4. Being ill takes meaning and purpose out of my life	3	2
	5. No preference	0	1
	Comment: Don't like any of the options. Suggestion: My illness makes it harder for me to make sense of life.	1	0
8	1. I think it best just to get on with life in spite of my changing/deteriorating health	3	3
	2. Despite my illness I try to focus on day to day life	5	4
	3. I interest myself in everyday things rather than think about my health	1	1
	4. No preference	0	1
9	1. It may not always feel like it but I do believe that I will come to accept the changes/deterioration in my health and its consequences	2	3
	2. I do believe I will come to accept my illness and its consequences	7	6

Some clear wording preferences for items 1, 2 and 9 emerged from the survey but for other items, respondents expressed a range of views. It was necessary to view the survey responses alongside the focus group discussions to provide a synthesis of the best fit with the theoretical concepts inherent in the scales, the palliative care context and linguistic clarity. Table 4 shows the original scale wording and the final revised version, alongside the Range of Response to Loss concepts. The items are grouped to reflect the three conceptual dimensions in the scale. The carer version of the scale uses equivalent wording eg Question 1**.** I am able to face up to the feelings I have about … 's illness. (revised version).

**Table 4.** Comparison Between the Original and Revised Versions of the Attitude to Health Change Scale, Patient Version.

Range of response to loss theoretical concepts	Original wording	Revised wording
Overwhelmed items		
Disturbingly intrusive thoughts	2. For me, it's difficult to switch off thoughts about the changes/deterioration in my health.	2. I find it difficult to switch off thoughts about my health.
Persistently painful emotions	5. I feel a constant sense of sadness about the losses caused by the change/deterioration in my health	5. I often feel emotional about my health eg fearful, anxious, sad…………
Life losing meaning	7. My changing/deteriorating health makes it harder for me to make sense of life.	7. My illness makes it harder for me to make sense of life.
Controlled items:		
A belief in stoicism	4. I believe that I must be brave in the face of my changing/deteriorating health.	4. I believe I should be brave when facing my illness.
Avoidance of expressions of distress	6. For me, it is important to keep feelings about my changing/deteriorating health under control.	6. It is important for me to keep my feelings about my health under control.
Diverting attention	8. I think it is best just to get on with life in spite of my changing/deteriorating health.	8. I try to focus on day to day life rather than my health.
Resilient items:		
Ability to face feelings	1. I am able to face the feelings which arise as my health changes/deteriorates.	1. I am able to face up to the feelings I have about my illness.
A sense of personal resourcefulness	3. I feel very aware of my inner strength when facing the consequences of the changes/deterioration in my health.	3. I feel emotionally strong enough to cope with my illness and its consequences.
Hopefulness/ positivity	9. It may not always feel like it but I do believe that I will come to accept the changes/deterioration in my health and its consequences.	9. I believe that I will come to accept my illness and its consequences.

## Discussion

The Attitude to Heath Change scales were developed by articulating the conceptual dimensions in the Range of Response to Loss theoretical model^13^ around the overwhelmed, controlled and resilient constructs, and by reframing the wording used in the validated Adult Attitude to Grief bereavement scale,^14^ to represent the range of emotional and cognitive reactions and coping responses to life-changing illness. Specialist practitioners with experience of using the scales affirmed their relevance to palliative care practice,^18^ with respondents noting the engagement of patients and carers with both the scales quantitative function of assessment and the qualitative function of a facilitatedtherapeutic conversation. In this study practitioners reflected on the scales wording, exploring issues of face validity, which stimulated ideas about how items might be clarified and simplified while maintaining theoretical congruence with the underpinning model. The outcomes of this research are revised Attitude to Health Change scales (patient and family carer versions) that make sense to expert practitioners, ready for the next stages of validation with patients, and psychometric testing.

A large number of assessment tools are used in palliative care^4,25^ and some psychosocial elements may form part of a multidimensional assessment or be implied within generic psychological measures. More recent research has moved beyond psychiatric classifcation to identify more specific psychosocial factors in palliative care.^26^ A small number of scales provide helpful comparisons with the Attitude to Health Change scales^27^ and have conceptual parallels eg between the Range of Response to Loss concept of being overwhelmed, and demoralisation, helplessness-hopelessness, but there are still notable distinctions. In contrast to scales which are symptom based and/or psychological extrapolations, the Attitude to Health Change scales constructs are theoretically based. The two dimensions of the Range of Response to Loss model provide the theoretical link between instinctive expressions of loss and conscious coping with loss. Support for this link is taken from the theoretical work of Mikulincer and Florian^16^ who connect attachment styles, the inheritantly acquired characteristics accrued from learning and experience, with emotional and cognitive reactions to stressful events. The specific manifestations of these characteristics are represented in the overwhelmed and controlled items in the scales along with the characteristics of resilience articulated by Greene^17^ in his study of Holocaust survivors and Seligman^1^ on the psychology of building human strength (See Table 1). The connectedness of all the items in the scales through the Range of Response to Loss model to these wider concepts of loss suggest their pertinence to palliative care.

The emphasis on resilience also makes the scales distinctive by recognising that even with evidence of distressing reactions to life limiting illness, resilience can be a mediating factor. While there has been increasing literature on promoting resilience in palliative care practitioners,^30^ and addressing the support needs of carers from a resilient perpsective,^33^ there has been less focus on resilience in patients.^34^

Refining the scales wording has made their constructs clearer for the practitioners who would be guiding the use of these scales in clinical practice and potentially more user-friendly for patients and carers. However, the focus groups highlighted other practitioner issues that need addressing including a focused Attitude to Health Change scales protocol and training.^35^ These need to be in place to ensure the scales are used ethically and proficiently in practice. Clinically, it is likely that the scales will be used for initial assessment and for ongoing review during illness progression The discursive function provides an engagement between patient/carer and practitioner, potentially deepening the insights both have about vulnerabilities in coping. This may show the need for, and focus required, for appropriate supportive interventions.

### Strengths and Limitations

The study sample was small and was restricted to practitioners who already had experience of using the Adult Attitude to Grief scale and who worked predominantly in specialist psychosocial care. Future work needs to explore the potential wider use of the scale by palliative care practitioners not specialist in psychosocial care. The study did not include the perspectives of patients and carers and exploring these is the next planned stage of scale development, which may result in further changes to the scales wording prior to full psychometric testing.

## Conclusion

The scales provide an opportunity to explore the impact of current life-changing illness, and all its consequent circumstances, alongside the predisposing factors which shape a person's attitudes and propensity for vulnerability and resilience. Patient and carer versions of the scale gives importance to this relationship dynamic and its effect on the different or even conflicting attitudes each bring to illness and palliative care. Using the scales as a framework for revealing the dynamic between patient and carers adds extra potential for person centred working. This work provides a sound base for future testing with patients and carers and for the scales’ psychometric validation.
